# Anatomically reduced fixation should always be considered when treating B and C proximal epiphyseal humeral fractures

**DOI:** 10.1186/s10195-022-00668-1

**Published:** 2022-11-05

**Authors:** Luigi Tarallo, Gian Mario Micheloni, Andrea Giorgini, Martina Lombardi, Beatrice Limone, Fabio Catani, Giuseppe Porcellini

**Affiliations:** Department of Orthopaedic Surgery, University of Modena and Reggio-Emilia, Via del Pozzo 71, 41125 Modena, Italy

## Abstract

**Background:**

Proximal humeral fractures are commonly observed in elderly patients. Management of these injuries is controversial. Literature comparing locking plate fixation, arthroplasty, and conservative treatments show no clear advantages for any of these management strategies. Thus far, no study has considered anatomically reduced fractures obtained after locking plate treatment. To clarify the best surgical procedure in middle-aged patients, we considered outcomes and major complications leading to surgical revision following an anatomically reduced fracture fixed with locking plate and reverse shoulder arthroplasty (RSA) in the treatment of type B/C fractures in patients between 50 and 75 years of age.

**Methods:**

This is a retrospective study including 59 patients between 50 and 75 years of age with type B/C proximal humeral fracture treated with RSA or with locking plate fixation (resulting in an anatomical reduction) between January 2010 and December 2018. Preoperative radiographs and computed tomography (CT) were evaluated in all patients. Clinical and radiologic follow-up was performed using range of motion (ROM), the Constant–Murley Score (CMS), the Oxford Shoulder Score (OSS), the Simple Shoulder Test (SST), the Subjective Shoulder Value (SSV), and visual analog scale (VAS). Major complications were considered.

**Results:**

In the plate fixation group, ROM, CMS, SST, and VAS were higher than in the RSA group. Lower complication rates compared with the literature were observed in both groups. Anatomically reduced fracture fixed with plate and screw could outperform RSA in terms of outcome. In second-level centers where traumatology is performed by surgeons with great expertise in upper limb trauma, the choice between plate fixation and reverse arthroplasty should be made during surgery.

**Conclusion:**

Anatomically reduced fractures showed better outcomes compared with RSA in type B/C fractures. Surgeons should always try to perform a reduction of the fracture in order to understand if a plate fixation could be feasible. If it is impossible to perform an anatomical reduction, we suggest to consider RSA. This is a retrospective observational study.

## Introduction

Proximal humeral fractures are the third most common osteoporotic fracture type observed in elderly patients, after wrist and hip fractures [1, 2]. Their incidence is 6–8%, with incidence peaks in the 60–90-year age groups and a female to male ratio of 70:30 [3]. Management of these common injuries is often challenging and controversial [4]. Patients are most commonly treated nonoperatively, but some complex patterns, mainly in younger people, require surgery [5]. The choice of surgical treatment for displaced fractures with percutaneous techniques, intramedullary nailing and locking plates, should consider the patient’s level of independence, bone quality [6], and risk factors.

In case of four-part fractures in elderly patients, reverse shoulder arthroplasty (RSA) is recommended [7].

Locking plate technology such as reduced friction and polyaxial locking screw positioning showed promising advantages [8], leading to a significantly increased use in the management of these fractures. Unfortunately, complication rates, including technical mistakes (e.g., intraarticular screw positioning, screw loosening) as well as loss of reduction, screw cut-out, head necrosis and non-union, were considerably high [9, 10]. The overall complication rate in patients > 60 years was reported to be as high as 45%, leading to revision surgery in 18% of the patients within 1 year [11].

In the literature, the treatment of three- or four-part proximal humeral fractures with RSA leads to good and predictable outcomes in people over 65 years of age, but there are no clear advantages compared with locking plate fixation, arthroplasty, or conservative treatment [12–14].

The main focus remains on the treatment choice in people with complex fractures patterns between 50 and 75 years of age, which represent a “gray zone” between fixation and arthroplasty.

As far as we know, no retrospective study has considered only anatomically reduced fractures obtained after locking plate treatment, and thus there could be a bias in evaluating the real outcomes of locking plates. We consider a reduction anatomic when the calcar part is restored, the neck–shaft angle is nearly 135°, and the tuberosities are in the native position.

To clarify which surgical procedure should be preferred, the purpose of our study was to assess patient outcome as well as the major complications leading to surgical revision following an anatomically reduced fracture fixed with locking plate and RSA in the treatment of type B and C fractures [according to AO Foundation/Orthopaedic Trauma Association (AO/OTA) classification] in patients between 50 and 75 years of age.

## Materials and methods

This is a retrospective study including 59 patients between 50 and 75 years of age with type B and C proximal humeral fracture, according to AO/OTA classification, treated with anatomical reduction and locking plate fixation (group I) or RSA (group II) between January 2010 and December 2018.

Exclusion criteria were other homolateral upper limb fractures, neurological injury due to trauma, fracture dislocations, split-head patterns, non-anatomical reduction of the fracture, and polytrauma.

**Fig. 1:**
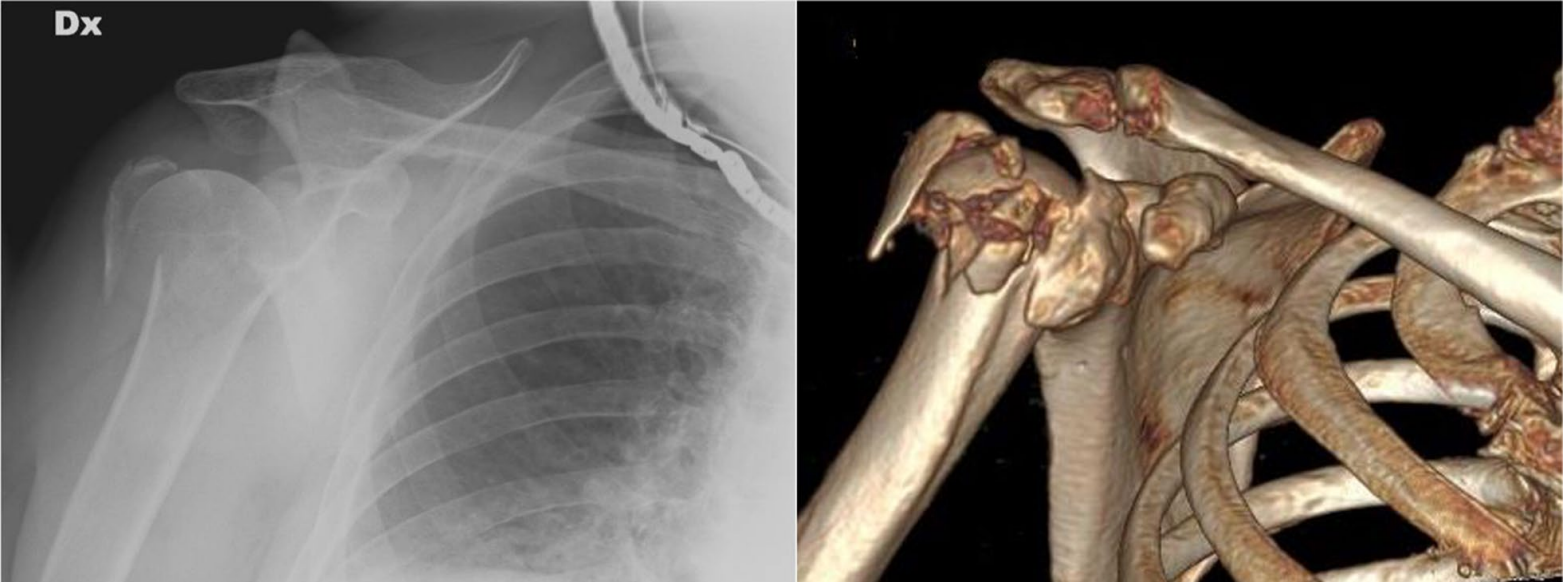
Preoperative X-ray and CT scan of proximal humeral fracture treated with plate fixation. **A** AP view; **B** tridimensional reconstruction using CT scan (group I)

**Fig. 2:**
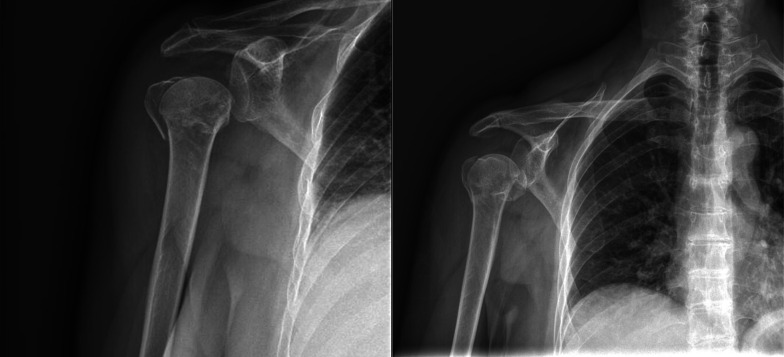
Preoperative X-ray of proximal humeral fracture treated with reverse shoulder arthroplasty (group II)

Preoperative standard radiographs of the shoulder (anterior–posterior view and *Y*-view orthogonal to the anterior–posterior view) and additional computed tomography (CT) were evaluated (Figs. 1, 2).

CT scans were evaluated in all patients to properly identify the fracture patterns, the position of the bone fragments, and the direction of the fracture lines.

**Fig. 3:**
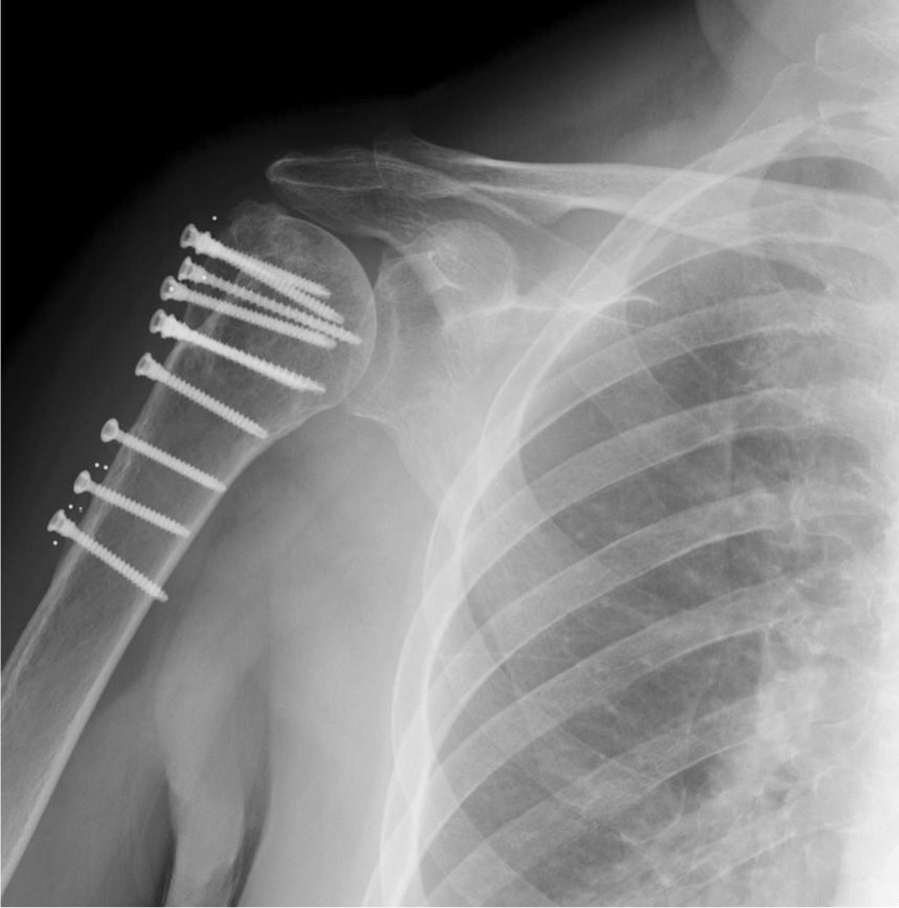
Postoperative X-rays after plate fixation

**Fig. 4:**
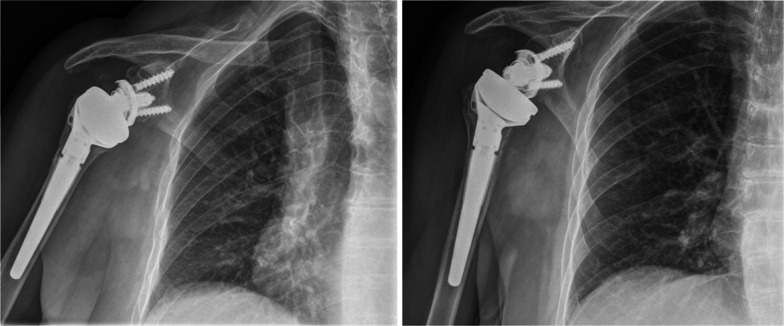
Postoperative X-rays after reverse shoulder arthroplasty

On follow-up, range of motion (ROM), the Constant–Murley Score (CMS), the Oxford Shoulder Score (OSS), the Simple Shoulder Test (SST), the Subjective Shoulder Value (SSV), and visual analog scale (VAS) were assessed. Postoperative X-rays were evaluated in all patients, in particular in group I to exclude non-anatomically reduced fractures after plate fixation (Fig. 3, 4). Major complications such as stiffness, impingement, osteonecrosis, and revision surgery were assessed by reviewing the electronic medical record of each patient.

### Group I—Plate fixation

All patients were treated with DiPhos (Lima Corporate, San Daniele del Friuli, Italy) LCP plate.

**Fig. 5:**
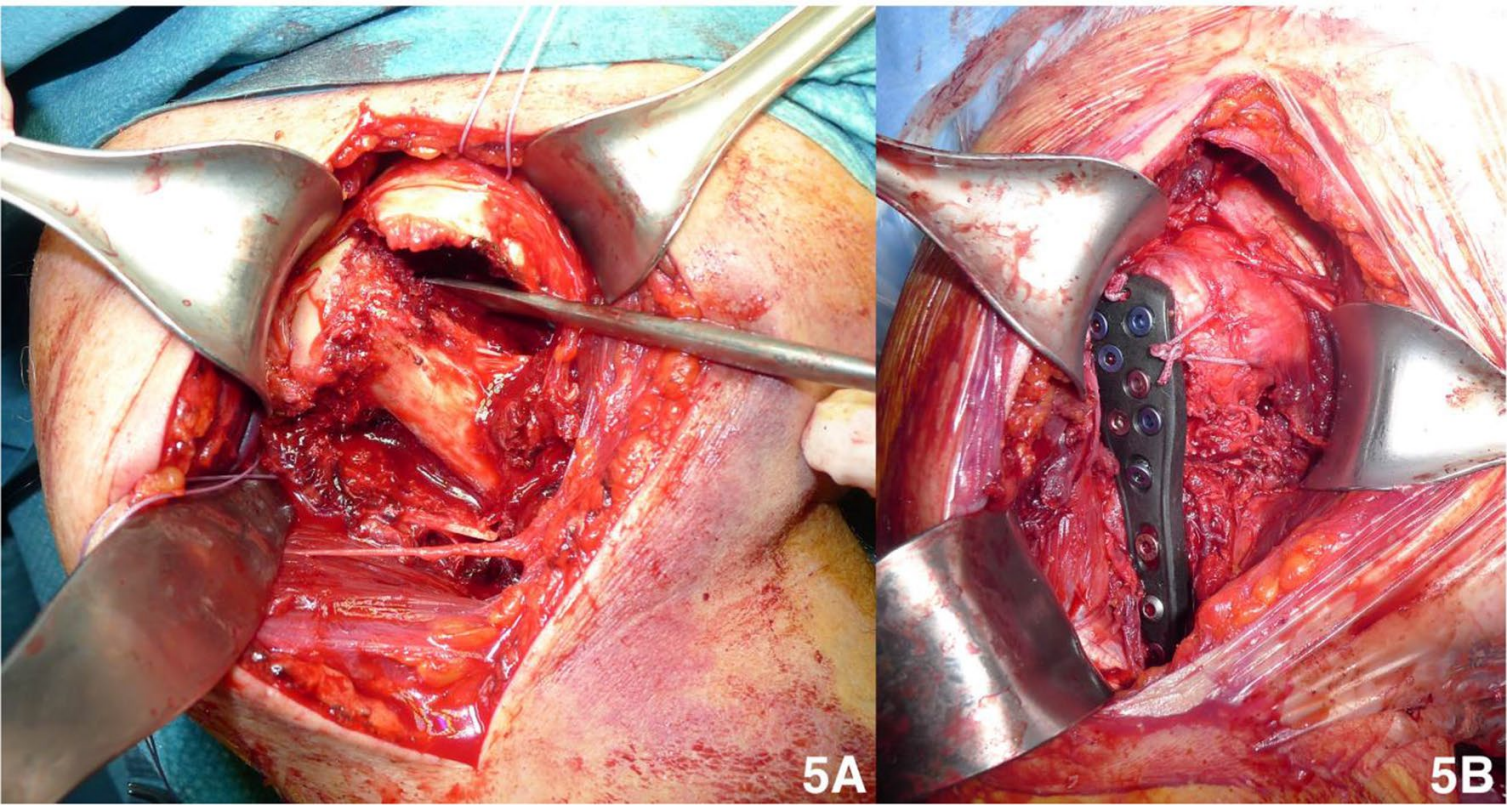
Intraoperative views of plate fixation surgery. **A** Assessment of bony fragments; **B** final results of osteosynthesis with reduction of the tuberosities

A deltopectoral approach was performed in all cases; the separated tuberosities were fixed with tension band sutures to reduce the free tuberosity fragments. Subsequently, the humeral head was reduced, and in case of medial metaphyseal fragmentation, the medial column was restored to avoid varus collapse. If necessary, the fracture reduction was temporarily secured using K-wires. Finally, the plate was fixed to the humeral shaft and head and the tuberosity sutures were passed through the small holes of the plate to fix the tuberosities (Fig. 5). In two cases, an intramedullar augmentation was performed with a fibular bone allograft because of the very poor metaphyseal bone quality. Biceps tenodesis was performed at the end of the procedure in all cases.

Postoperatively, the arm was immobilized in a sling for 4 weeks. The patients were allowed to start physiotherapy after 15 days: passive-assisted abduction and flexion were restricted to 90° for the first 4 weeks. After 1 month, active exercises and rotations were allowed.

### Group II—RSA

Patients of this group were treated with SMR system (Lima Corporate, San Daniele del Friuli, Italy) uncemented reverse arthroplasty.

**Fig. 6:**
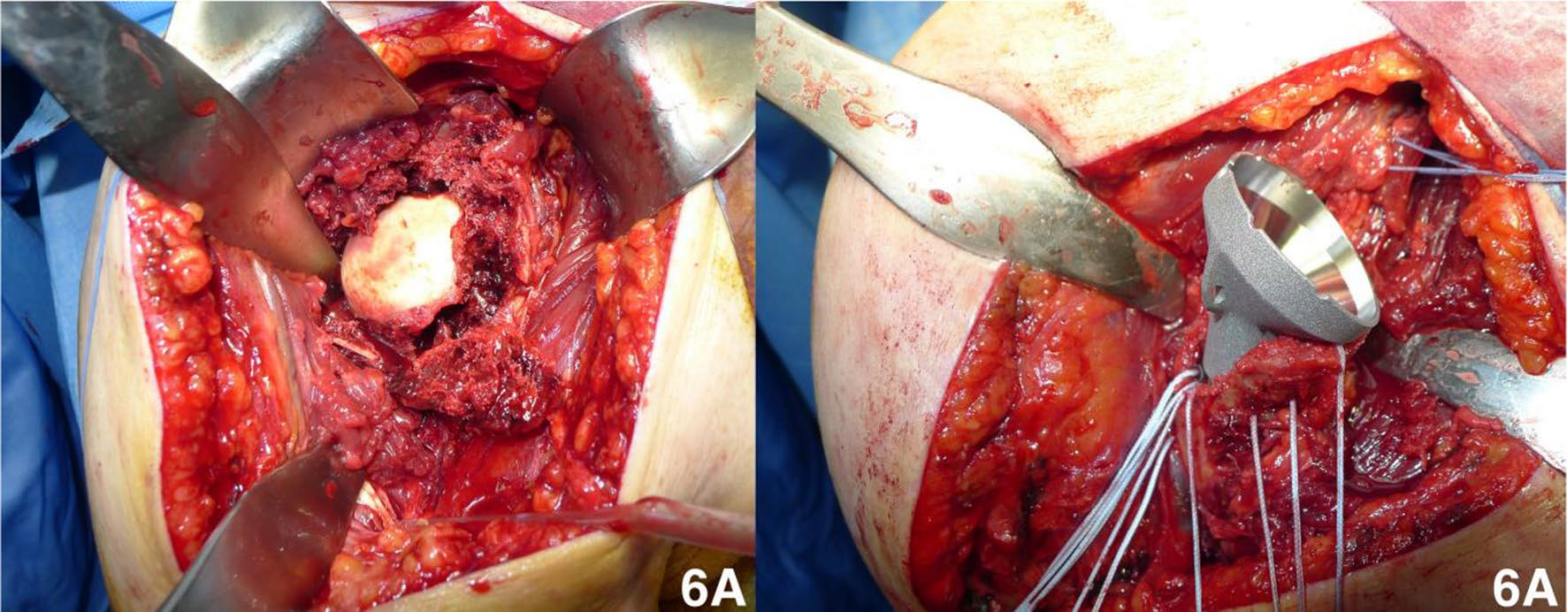
Intraoperative views of replacement surgery. **A** Assessment of bony fragment with humeral head involvement; **B** humeral stem positioning with tuberosities fixed to the diaphysis and to the stem with high-resistance Fiberwire sutures

Deltopectoral approach was performed. Tuberosities were appositely prepared for later fixation to the humeral stem and humeral shaft. The humeral head and all loose bone fragments were removed (Fig. 6). The glenoid was exposed and prepared for implantation of the baseplate and of the polyethylene glenosphere. The humeral shaft was then exposed. The intramedullary cavity was prepared with progressive broaches, and four holes were performed in the shaft for the final tuberosities reduction. The definitive prosthesis stem with metal liner was placed and reduced, testing the correct soft tissue tension. In all cases, the tuberosities (or part of them) were fixed to the diaphysis and to the stem with high-resistance Fiberwire (Arthrex, Naples, Florida) sutures to improve stability and function of the arthroplasty (Fig. 6).

All patients wear a sling for 4 weeks. Passive-assisted motion was allowed from the first postoperative day, and active motion was allowed from the third week. Shoulder internal and external rotations were restricted for the first 6 weeks.

#### Subanalysis

Subgroup analysis was performed on the basis of type of fracture, comparing patients with type C pattern according to AO/OTA classification with the whole population.

### Statistic

Statistical analysis was made with unpaired *t*-test to assess significant differences between the two groups. A *p* value < 0.05 was considered statistically significant. Statistics were calculated using commercially available programs (IBM SPSS Statistics for Windows, Version 22; Armonk, NY, USA).

## Results

Fifty-nine patients [49 female (F), 10 male (M)] were enrolled in our study following the inclusion criteria. Forty patients (31 F, mean age 64.9 ± 6.4 years; 9 M, mean age 63.2 ± 5.9 years) were allocated to the plate fixation group with anatomical reduction (group I) and 19 patients (18 F, mean age 68.4 ± 6.8 years; 1 M, age 67 ± 5.6 years) to the RSA group (group II). Minimum follow-up was 24 months (mean 58 months). Preoperative CT scans allow us to classify 20 type B fractures and 20 type C fractures in group I, 16 type C fractures and 3 type B fractures in group II, according to AO/OTA classification.

**Fig. 7:**
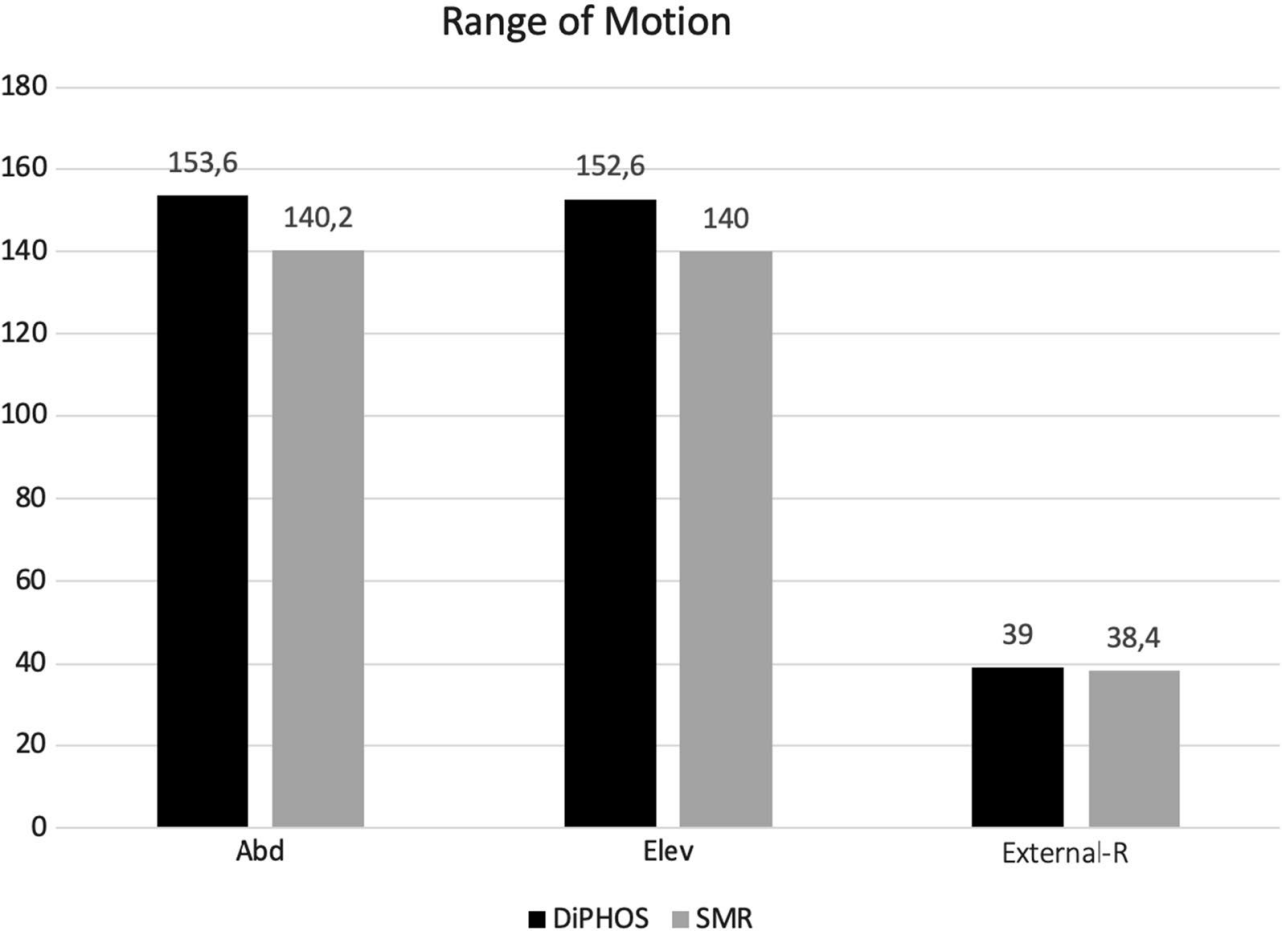
Diagram: ROM comparison between group I and group II. *Abd* abduction, *Elev* elevation, *External-R* external rotation

Group I showed a better trend in functional outcomes regarding ROM mainly in internal rotation, while the external rotation was similar in both groups (Table 1, Fig. 7), without reaching statistical significance.

**Table 1:** Analysis of the total population.* CSM* Constant–Murley Score,* OSS* Oxford Shoulder Score,* SST* Simple Shoulder Test,* SSV* Subjective Shoulder Value,* VAS* visual analog scale

Total population	Total (*n* = 59)	DiPHOS (*n* = 40, 67.8%)	SMR (*n* = 19, 32.2%)	*p* value
Age, years	66 ± 6.2 (50–75°)	64.1 ± 6.7 (50–75°)	68.8 ± 4.4 (60–75°)	
Abduction	149.3 ± 28.5° (60–180°)	153.6 ± 23.8° (90–180°)	140.2 ± 35.6° (60–180°)	0.093
Elevation	148.5 ± 27.8° (80–180°)	152.6 ± 25.9° (80–180°)	140 ± 32.1° (90–180°)	0.103
External rotation	38.8 ± 11.7° (10–60°)	39 ± 10.8° (20–60°)	38.4 ± 13.8° (10–60°)	0.861
Internal rotation	L3	T10 (T7–buttock)	Sacrum (T7–buttock)	
CMS	70.8 ± 19.9° (26–99°)	77.3 ± 18.9° (30–99°)	57.1 ± 14.6° (26–79°)	< 0.001
OSS	41.0 ± 9.1° (13–52°)	42.3 ± 8.4° (20–52°)	38.1 ± 9.9° (26–79°)	0.092
SST	8.9 ± 2.5° (2–12°)	9.7 ± 2.0° (4–12°)	7.1 ± 2.5° (2–11°)	< 0.001
SSV	0.7 ± 0.1° (0.3–1°)	0.8 ± 0.14° (0.4–1°)	0.7 ± 0.1° (0.3–0.95°)	0.071
VAS	2.0 ± 2.3° (0–8°)	1.45 ± 1.6° (0–7°)	3.3 ± 2.8° (0–8°)	0.002

The mean Constant–Murley score, Simple Shoulder Test, and VAS scale are significantly higher in group I compared with group II. The Oxford Shoulder Score and the Subjective Shoulder Value show better results in group I than in group II, even if they are not statistically significant (*p* = 0.092, *p* = 0.071).

With subgroup analyses, we selected only type C fractures to evaluate differences between the two groups: group II exhibited a trend for worse ROM, though it was not statistically different, while CMS (74.8 ± 20.4 in group I versus 57.1 ± 14.6 in group II), SST (9.4 ± 2.2 in group I versus 6.9 ± 2.5 in group II), and VAS (1.7 ± 1.9 in group I versus 3.5 ± 3 in group II) were significantly higher in the plate fixation group compared with the RSA group (Table 2).

**Table 2:** Comparison between type C fractures and total population

Type C	Total (*n* = 36)	DiPHOS (*n* = 20, 55.6%)	SMR (*n* = 16, 44.4%)	*p* value
Age, years	66.1 ± 6.2 (50–75)	64.1 ± 6.7 (50–75)	68.8 ± 4.4 (60–75)	
Abduction	144.8 ± 31.6° (60–180°)	151 ± 25.5° (90–180°)	137.1 ± 37.4° (60–180°)	0.197
Elevation	145.8 ± 29.3° (90–180°)	150 ± 26.3° (80–180°)	136.8 ± 31.2° (90–180°)	0.102
External rotation	37.2 ± 12.0° (10–60°)	38 ± 11.0° (20–60°)	36.2 ± 13.6° (10–60°)	0.672
Internal rotation	L2	T10 (T7–buttock)	Sacrum (T7–buttock)	
CMS	66.2 ± 20.3 (26–99)	74.8 ± 20.4 (31–99)	57.1 ± 14.6 (26–74)	0.003
OSS	39.6 ± 10.4 (13–48)	40.9 ± 10.3 (20–48)	38.1 ± 10.6 (13–48)	0.425
SST	8.3 ± 2.6 (2–12)	9.4 ± 2.2 (4–12)	6.9 ± 2.5 (2–11)	0.004
SSV	2.5 ± 2.6 (0–8)	0.8 ± 0.15 (0.4–1)	0.7 ± 0.1 (0.3–0.95)	0.281
VAS	2.0 ± 2.3 (0–8)	1.7 ± 1.9 (0–7)	3.5 ± 3 (0–8)	0.034

Regarding complications in plate fixation group (Table 3), we recorded five patients with restricted ROM (12.5%) and one osteonecrosis (15%). Impingement occurred in one patient (2.5%). The revision surgery rate was 12.5%. The plate was removed in five patients (12.5%).

**Table 3:** Complications in plate fixation group

Complications	DiPHOS (*n* = 40)
Stiffness	5 (12.5%)
Impingement	1 (2.5%)
Osteonecrosis	1 (2.5%)
Revision surgery	5 (12.5%)

Screw cutout, pseudoarthrosis, and malunion were avoided in all cases.

In the RSA group, neither radiolucency nor bone resorption was recorded around the stem. Patients of this group did not require any revision surgery.

## Discussion

**Fig. 8:**
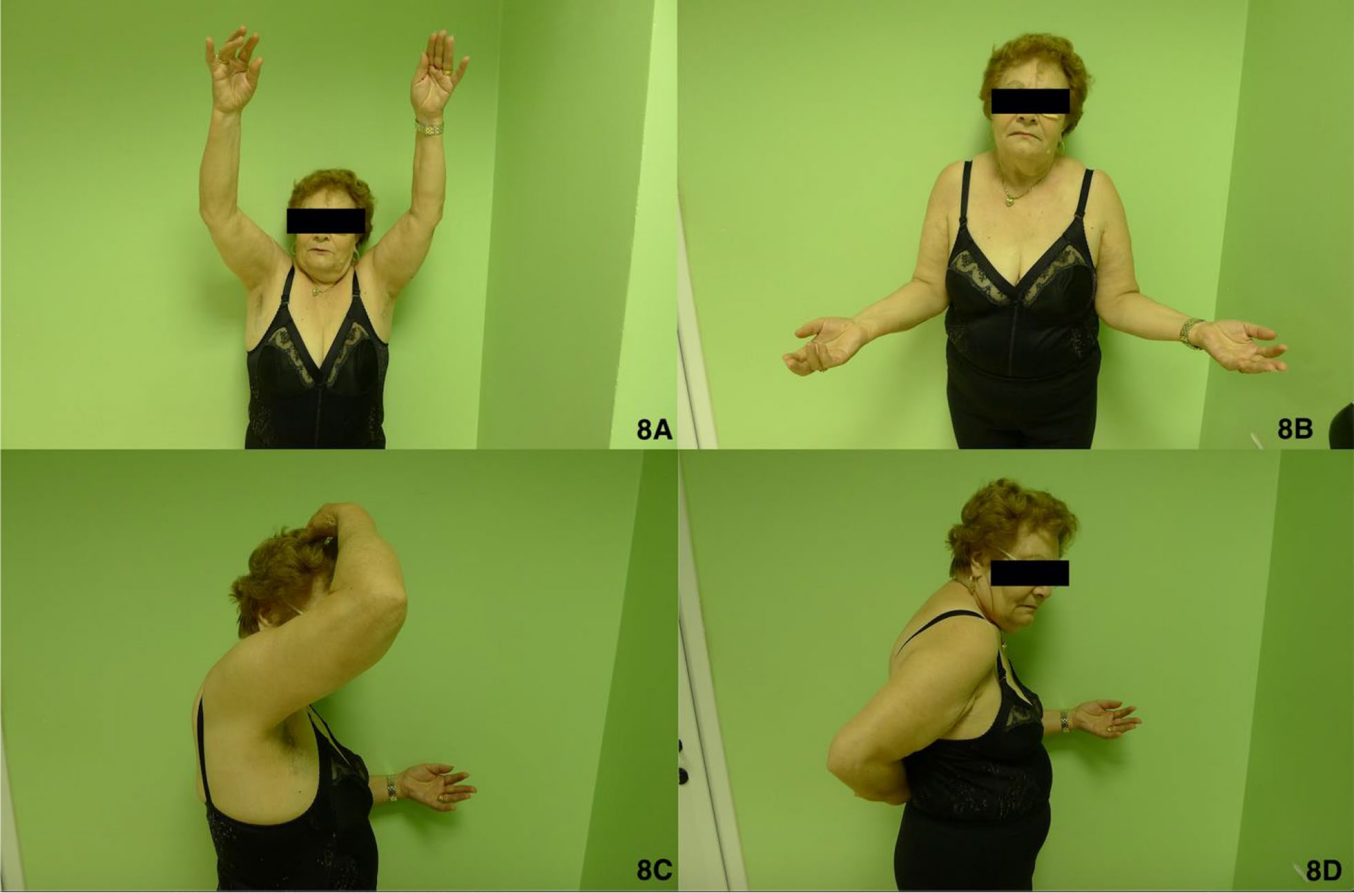
ROM of a group I 70-year-old female. **A** Elevation; **B** external rotation; **C** abduction; **D** internal rotation

**Fig9:**
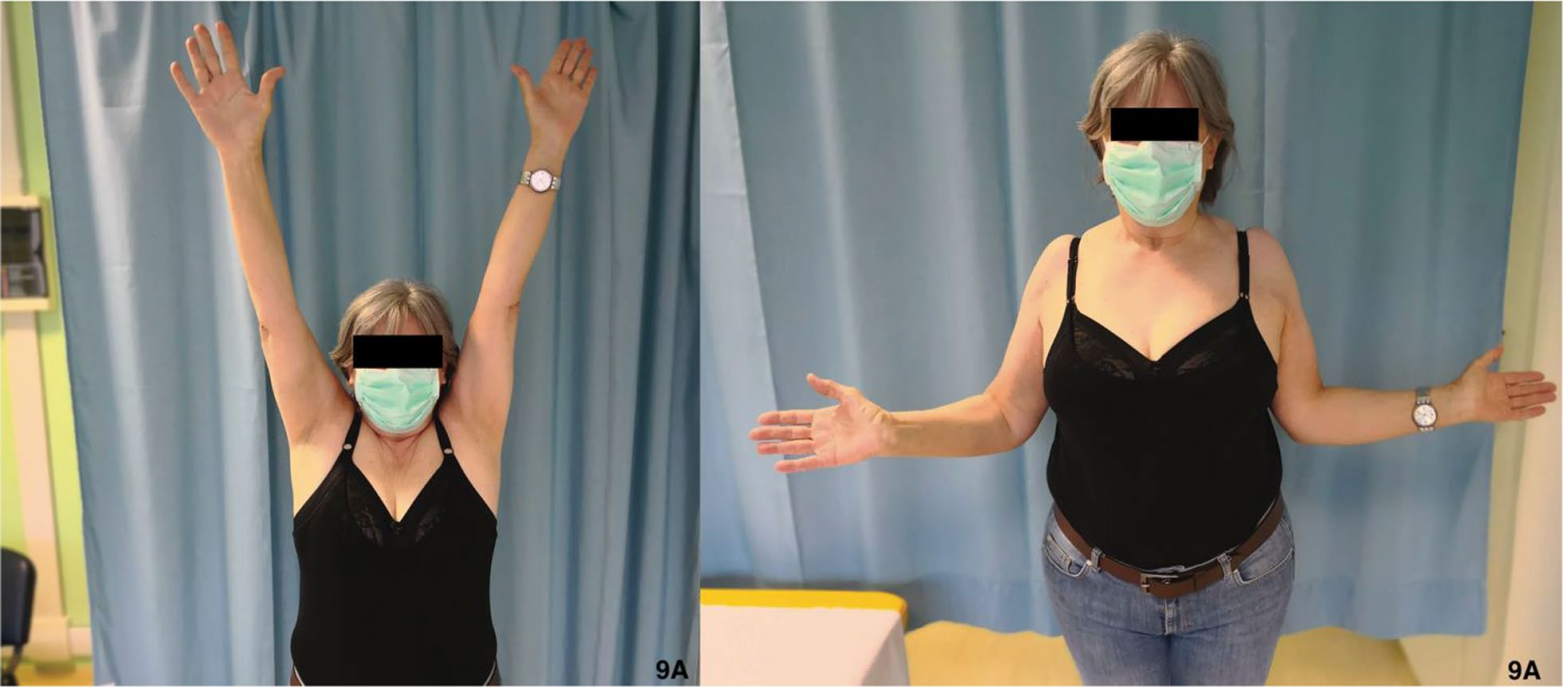
ROM of a group II 69-year-old female. **A** Elevation; **B** external rotation

In the present study, in the plate fixation group ROM was slightly higher than in the RSA group (Table 1, Fig. 5), although the difference did not reach statistical significance. Considering postoperative intrarotation, which depends largely on the correct healing of the tuberosity around the prosthetic implant [15], group I showed better results: these patients reached T10 as the maximum level, compared with sacrum level reached by the RSA group (Fig. 8, 9). The extrarotation values were not significantly different (*p* = 0.861).

This result can be explained by the surgical technique used during the procedure of osteosynthesis with plate and screw: the separated tuberosities were fixed with sutures to obtain an anatomical reduction as well as to adequately isolate it from the surrounding tissues. The aim is to fix the fragments at a correct height with respect to the plate and at the right tension to achieve correct healing of the bone [16–18]. If the tension is too high, the infraspinous and teres minor muscles displace the great tuberosity posteriorly and the subscapular muscle displaces the minor tuberosity medially, with loss of rotational movement of the shoulder.

The plate material can represent another reason explaining the better outcome obtained using this technique: the CFR PEEK 30% is a polymer that allows one to observe the intraoperative reduction and follow the imaging evaluation without CT or MRI artifacts. This inert material allows the muscles (deltoid) to slide on the plate more than titanium, with better ROM outcomes [19]. The lower risk of bone ingrowth on the plate is another advantage of this material.

The CMS showed good results in both groups, with a score of 77.3 ± 18.9 for the plate group, significantly better than the results obtained for RSA (57.1 ± 14.6). SST (9.7 ± 2.0) and VAS (1.45 ± 1.6) were also higher in group I (*p* < 0.001 and *p* = 0.002, respectively).

It is well known that bone quality worsens with increasing age, giving rise to more complex fracture patterns [20]. Nevertheless, subgroup analysis of type C fractures was applied to a subpopulation whose age in both groups was comparable to the entire population considered for the study (Table 2).

Clinical and functional outcomes were slightly worse than in the entire population, except for intrarotation (Table 2). A significant superiority of CSM, SST, and VAS was confirmed in the plate group compared with the RSA group.

Fraser et al. [21] showed better results concerning CMS and OSS in the RSA group. The study otherwise suggested that prosthetic replacement may have better outcomes than plate fixation in the type B/C fractures treatment in elderly population. Chalmers et al. reached the same conclusion regarding the superiority of RSA over synthesis and HA (hemiarthroplasty) [16].

Gallinet et al. also demonstrated that RSA revealed higher clinical and functional recovery and greater pain control [22].

The meta-analysis of Walters et al. [23] concluded that the surgical choice should consider the age of the patient, the level of independence, the bone quality, the comorbidities, and the fracture pattern. Healthy patients with complex pattern fractures are candidates for surgical synthesis with plate and screws; HA should be reserved for patients with poor bone quality and pattern fractures that might lead to AVN (high risk of cutout screws); RSA should be considered for elderly patients, maybe after a first attempt with ORIF or HA [8, 24].

Our results are considerably different from those of previous studies that consider all fractures treated with locking plate, without any selection regarding the reduction obtained after fixation [16].

The present study showed that anatomically reduced fracture fixed with plate and screw could overcome RSA outcomes; therefore, in a second-level center where traumatology is performed by surgeons with great expertise in upper limb trauma, the choice between plate fixation and reverse arthroplasty should be made during surgery, after a direct evaluation of the reduction of the fracture.

A recent meta-analysis by Gupta et al. [25] confirmed better outcomes with plate fixation with respect to RSA, although a higher complication rate was recorded (15%) [26].

In contrast to literature [10], we recorded neither screw cutout nor loss of reduction in group I, while one case of osteonecrosis occurred. Four patients underwent surgical plate removal: two of them underwent open surgery to repair the rotator cuff approximately 2 years after surgery, and in two subjects removal of the plate was necessary because of humeral head AVN, followed by RSA implantation 1 year after surgery; one patient underwent arthroscopic arthrolysis for stiffness, associated with plate removal, 1 year after surgery.

The lower complication rate compared with the literature [27, 28] was probably due to a restriction in the inclusion criteria: anatomically reduced fractures could have lower complication rates.

The revision surgery rate was 10%, slightly lower than reported in the literature [10].

Aseptic or septic loosening [29], tuberosity resorption, or scapular notching did not occur in the RSA group. Revision surgery was never required.

Strength of the present study include the restrictive inclusion criteria, which allow authors to investigate the real outcome of a well-performed synthesis, and the exclusion of patients with non-anatomically reduced fractures. Limitations of this study include the mid-term follow-up, the small sample of patients, and the different amounts of patients in the two groups.

## Conclusion

Anatomically reduced fractures showed better outcomes with respect to RSA in type B/C fractures, even if a 10% rate of revision surgery was recorded. When treating these fractures, it would be wise to prepare the operating room to allow both synthesis and RSA to be carried out. To choose the best treatment, the surgeon should always try to perform a reduction of the fracture in order to understand whether a plate fixation could be feasible. If it is impossible to perform an anatomical reduction, we suggest to consider RSA.

## Data Availability

All data generated or analyzed during this study are included in this published article.
